# Local Anæsthesia with Cocaine and Adrenalin

**Published:** 1912-03-02

**Authors:** N. Stuart Twist

**Affiliations:** Leeds, late Senior Casualty House Surgeon, Hull Royal Infirmary.


### LOCAL ANÆSTHESIA WITH COCAINE AND ADRENALIN.

By N. STUART TWIST, M.B., Ch.B. Leeds, late Senior Casualty House Surgeon, Hull Royal Infirmary.

The great majority of surgeons fight shy of cocaine as a local anæsthetic. This is probably no doubt due to the large quantity of cocaine usually used, and the toxic effects consequent on this large dosage. Infiltration of the tissues is the principle which should guide in the use of cocaine as a local anæsthetic.

A solution of 0.5 per cent. of cocaine is perfectly satisfactory and almost absolutely non-toxic if used with 5 or 10 minims of a 1 in 1000 solution of adrenalin chloride added to each ounce of the solution. Such a solution can be easily prepared at the time of the operation by using powders of cocaine (made up of 2½ grains of cocaine hydrochloride). A dozen such powders, ready weighed, will cost about 1s. 3d.

By the addition of one of the powders to an ounce of water which has been boiled and allowed to cool to about the body temperature, and then adding about five drops of the adrenalin solution to this, the solution required is easily prepared. If more or less is required, a proportionate amount of the powder must be used.

Of such a solution one need have no fear of injecting an ounce or even more. Toxic effects are practically negligible. Two ounces and even more have been used by the writer for amputations, etc., on several occasions without the very slightest ill-effect. Although the principal effect of this solution as an anæsthetic is due to the infiltration, the cocaine certainly plays a part. A solution of quinine and urea is used as a local infiltrative anæsthetic, but does not appear to cause such complete anæsthesia as the cocaine solution.

It is as well that the injection should be given with the patient in the recumbent position. The only toxic effect I have ever seen was a slight and short syncopal attack, the operation being performed with the patient sitting up; although, on the other hand, several of the minor operations, in which the preparation was used, were performed with the patient sitting up, and in many cases watching and following every step with interest with not the slightest suspicion of faintness.

The depth of anæsthesia is very complete, practically every patient declaring that they felt nothing whatever during the whole time.

The skin or mucous membrane having been prepared by one of the usual methods for the operation, the thin needle is inserted just beneath the epidermis in a slanting direction and a small quantity of the solution is injected. The skin around the needle becomes white and anæsthetic; the needle may be slowly and gradually pushed deeper into the subcutaneous tissues without this being felt by the patient, and the solution then freely injected until the part becomes distended with the fluid.

The distribution of the injected solution will, of course, vary with the operation, but if a simple incision is required this is all that is necessary. There should be no interval between the injection and the operation. Local anæsthesia is perfectly satisfactory for all amputations of the toes and fingers, the most timid patients expressing delight at not being subjected to a general anæsthetic.

Far too many patients requiring simple minor operations are put to the inconvenience of a general anæsthetic, which, however carefully and slowly given, is unpleasant and a thing to be avoided if possible, and is the part which the patient usually dreads much more than the operation itself.

Cocaine and adrenalin anæsthesia, besides being suitable for all amputations of toes and fingers, may be used for all circumcisions where the patient is beyond infancy, practically all empyæmata, the removal of subcutaneous and cutaneous growths, such as sebaceous and dermoid cysts, ganglia, lipomata, adenomata of the breast, some cases of enlarged glands in the neck, and in the removal of foreign bodies from under the skin and subcutaneous tissues.

For digital amputations the best method is to inject freely around the joint at which the amputation is to be made—this is frequently all that is necessary—the distal parts almost immediately becoming anæsthetic. Occasionally this is not so, and those distal parts which will be actually incised have also to be infiltrated with the fluid before the anæsthesia is complete. There is no need to be afraid of this as practically none of the solution will be absorbed, for it will be removed together with the parts amputated.

A useful addition to prevent absorption is a constricting band at the base of the finger or toe (or penis in the case of circumcisions), and this at the same time helps to increase the infiltration and distension of the tissues.

For empyæmata this method of anæsthesia is very satisfactory.

I lately operated on a boy of six years of age suffering from a double empyæma. The left side was opened under chloroform anæsthesia, and although this was carefully induced and lasted for but a couple of minutes, he was very ill indeed after it and was for some time afterwards in a very critical condition.

The other side was opened under cocaine and adrenalin without any pain or disturbance to the boy whatever. This is, of course, only practicable when the patient is old enough to assist the surgeon by keeping as quiet as possible. In circumcisions this also applies. Another precaution that is necessary in this operation is that care should be taken that the mucous surface of the prepuce is anæsthetic. This is best done by injecting a few minims of a 5 per cent. solution with a glass syringe between the prepuce and glans and holding this in for a few seconds. In the removal of foreign bodies under the skin the injecting needle will frequently be of use in localising the foreign body.

